# The *PIN* Gene Family in Cucumber (*Cucumis sativus* L.): Genome-Wide Identification and Gene Expression Analysis in Phytohormone and Abiotic Stress Response

**DOI:** 10.3390/plants14111566

**Published:** 2025-05-22

**Authors:** Yongxue Zhang, Kaili Zhu, Weiyao Shen, Jiawei Cui, Chen Miao, Panling Lu, Shaofang Wu, Cuifang Zhu, Haijun Jin, Hongmei Zhang, Liying Chang, Xiaotao Ding

**Affiliations:** 1 Shanghai Key Laboratory of Protected Horticulture Technology, Horticultural Research Institute, Shanghai Academy of Agricultural Science, Shanghai 201403, China; zhangyongxue@saas.sh.cn (Y.Z.); z18253926832@163.com (K.Z.); 19542816801@163.com (W.S.); cuijiawei@saas.sh.cn (J.C.); miaochen@saas.sh.cn (C.M.); lpl2245@163.com (P.L.); sfwu@saas.sh.cn (S.W.); zhucuifang1996@163.com (C.Z.); jinhaijun@saas.sh.cn (H.J.); zhanghongmei@saas.sh.cn (H.Z.); 2 College of Ecological Technology and Engineering, Shanghai Institute of Technology, Shanghai 201418, China; 3 College of Life Sciences, Shanghai Normal University, Shanghai 200234, China; 4 School of Agriculture and Biology, Shanghai Jiao Tong University, Shanghai 200240, China; changly@sjtu.edu.cn

**Keywords:** PIN, auxin, cucumber, hormone, abiotic stress

## Abstract

The auxin efflux transporter PIN protein plays a crucial role in the asymmetric distribution of auxin on the plasma membrane, influencing the growth and development of plant organs. In this study, we identified nine members of the *PIN* gene family in the cucumber genome, which could be classified into five phylogenetic groups. These genes have diverse structures but conserved transmembrane domains. Analysis of cis-acting elements in the promoters revealed that *CsPINs* contain 48 types of cis-acting elements, predominantly light-responsive elements and plant hormone response elements. In addition, PIN proteins may interact with a variety of auxin-related proteins (including auxin response factor, auxin binding protein, mitogen-activated protein kinase PINOID, etc.) to jointly regulate the auxin synthesis and metabolic pathways. We analyzed the expression profiles of *PIN* genes in 23 tissues of cucumber using the CuGenDB database, and further investigated the expression levels of *PIN* genes in leaves and roots in response to different abiotic stresses and hormone treatments by qRT-PCR. This study provides a theoretical basis for clarifying the regulatory mechanism of the cucumber *PIN* gene family during environmental stress processes.

## 1. Introduction

Auxin, as a small signaling molecule in plants, mainly exists in the form of indole-3-acetic acid (IAA). Auxin is widely distributed in various plant tissues and organs and participates in multiple biological functions, such as establishing the apical–basal polarity during embryogenesis, forming apical and axillary meristems, promoting fruit ripening, shaping root system architecture, and influencing plant tropisms [1,2]. Auxin is mainly synthesized in the apical meristem, rapidly generating a concentration gradient and is transported to different organs through polar auxin transport mechanisms [3]. The polar transport of auxin between plant cells is mainly mediated by three transporter families, including the auxin resistance 1/auxin-like 1 (AUX/LAX), the ATP-binding cassette B/P-glycoprotein (ABCB), and the PIN-forming (PIN) influx carrier [4]. Members of these gene families control the influx and efflux of auxin, forming a complex auxin regulatory network that regulates growth and development and responses to environmental stimuli [5,6,7].

With the advancement of next-generation sequencing technology [8], PIN family members have been identified in multiple plant species. Specifically, 8, 10, 10, 11, 11, 11, 12, 14, 15, 17, 20, and 23 *PIN* family members have been identified in the genomes of *Arabidopsis* [9], pepper (*Capsicum annuum*) [10], potato (*Solanum tuberosum*) [11], maize (*Zea mays* L.) [12], *Medicago truncatula* [13], *Sorghum bicolor* [14], grapes (*Vitis vinifera*) [15], pear (*Pyrus bretschneideri*) [16], poplar (*Populus trichocarpa*) [17], cotton (*Gossypium hirsutum*) [18], tobacco (*Nicotiana tabacum*) [19], and soybeans (*Glycine max*) [20], respectively.

To date, the gene functions of several members of the *PIN* family have been characterized in specific crop species. Plasma membrane (PM)-localized AtPIN1-4/7 mediate directional auxin transport through long central hydrophilic loops [21,22], whereas endoplasmic reticulum (ER)-localized AtPIN5/8 regulate intracellular homeostasis via short loops [23,24,25]. AtPIN6 uniquely dual-localizes to PM/ER, indicating that it may be involved in both intercellular auxin transport and homeostasis regulation [26,27]. Key *Arabidopsis* PIN proteins orchestrate developmental processes through specialized functions: AtPIN1/8 regulate floral development via embryonic auxin gradients (AtPIN1) and pollen-specific gametogenesis (AtPIN8) [23,28,29,30]. AtPIN2-5 mediate root architecture, with AtPIN2 governing meristem elongation, AtPIN3/4 coordinating apical hook formation, and AtPIN5 driving lateral root initiation [6,25,30,31,32]. AtPIN6 uniquely modulates multi-tissue development, including apical dominance and nectar formation [26,33,34]. AtPIN7 establishes embryonic polarity through basal PM-localized auxin maxima [35]. These regulators establish auxin distribution patterns critical for organogenesis. OsPIN1a/b regulate root system development, with overexpression enhancing lateral root formation [36]. OsPIN2 modulates shoot-to-root auxin redistribution [37], while OsPIN9/10s potentially drive adventitious root initiation [38]. ZmPIN1a/b mediate auxin transport during maize embryogenesis and endosperm development, with ZmPIN1a exhibiting sustained upregulation throughout stem maturation [39,40]. In addition, under drought, salt, and cold stress, most *ZmPIN* genes are induced to express in the stems of maize, while their expression is inhibited in the roots [41].

Cucumber (*Cucumis sativus* L.) is one of the most economically important vegetable crops worldwide. However, genome-wide information on *CsPIN* family members has not been reported. Auxin polar transport forms concentration gradients and local differences through the synergistic effect of synthetic and metabolic pathways, thereby achieving precise regulation of plant growth and development, tropism, and responses to endogenous and exogenous signals. In this study, we identified nine *CsPIN* genes and classified them into five groups. Furthermore, the physicochemical properties, phylogenetic relationship, chromosome localization, collinearity analysis, gene structure, cis-acting elements, and protein interaction prediction were comprehensively analyzed. The transcriptional levels of *CsPIN* genes in various tissues/organs and under abiotic stress conditions were analyzed through gene expression profiling and qRT-PCR. The results of this study will provide a theoretical basis for analyzing the function of the *PIN* gene in cucumbers and lay the foundation for the breeding of new high-quality cucumber varieties.

## 2. Results

### 2.1. Genome-Wide Identification and Phylogenetic Analysis of PIN Proteins in Cucumber

Using eight PIN protein sequences of *Arabidopsis* as the queries, a local Blastp (E-value 1 × 10^−5^) search was performed in the cucumber genome, and 68 protein sequences were obtained initially. A total of 15 candidate genes were identified according to conserved domain (PF03547). After the combined sequences were duplicated, 73 candidate genes remained. Then, SMART and CDD databases were used to predict the domains of candidate genes, and nine *CsPIN* genes were eventually identified. Moreover, gene locus, chromosome location, open reading frame length, and physical and chemical properties of *PIN* gene family members were analyzed, as shown in Table 1. The gene members have a length of 356 to 645 amino acids (aa), molecular weights ranging from 38.98 to 70.83 kDa, and a theoretical isoelectric point (pI) in the range of 7.04 to 9.59. Aliphatic index and hydrophilicity predicted that CsPIN is a mostly hydrophobic protein. CsPINs are multiple transmembrane proteins, which are mainly predicted to be located in PM and ER.

### 2.2. Phylogenetic Relationship Analysis of Cucumber PINs

**Figure 1:**
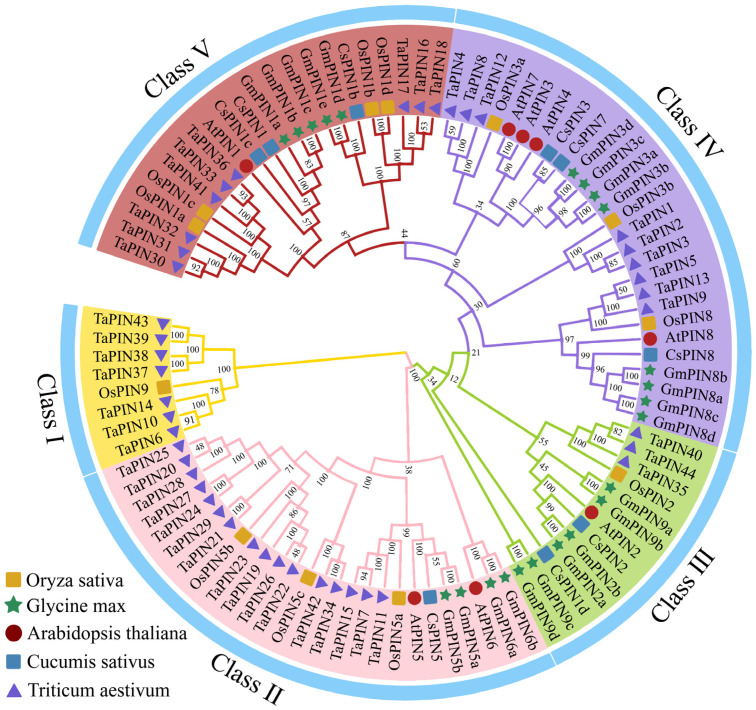
The phylogenetic tree of the PIN proteins from five species. The subgroups were designated through comparative analysis with *O. sativa* (12, yellow squares), *G. max* (23, green stars), *Arabidopsis* (8, red circles), and *T. aestivum* (44, purple triangles).

To further understand the phylogenetic relationship between the *CsPIN* gene family of cucumber and *PIN* genes of other species, we constructed a phylogenetic tree using PIN protein sequences of *Arabidopsis* (8), wheat (44), soybean (23), rice (12), and cucumber (Figure 1). They were divided into five subgroups based on sequence similarity and labeled with different colors. Group II contained one *CsPIN5* gene. The third group contains two genes, *CsPIN1d* and *CsPIN2*. Groups IV and V each contained three cucumber PIN family members (*CsPIN1*/*1b*/*1c* and *CsPIN3*/*7*/*8*). The aggregation of PIN protein sequences into five groups indicates that they have similar functional or subfunctional roles in species-dependent development. In group IV, cucumber *PIN* genes were clustered with *Arabidopsis* homologs (e.g., PIN3, PIN7, PIN8). In Arabidopsis, PIN3 has been shown to regulate hypocotyl gravitropism by redistributing auxin in root columella cells [42], while PIN7 mediates light-induced phototropism through asymmetric auxin transport in hypocotyls [43]. Additionally, *pin8* exhibits impaired root gravitropic bending and shoot phototropic responses [44]. These results suggest that PIN family proteins are evolutionarily conserved and play a key role in mediating auxin-dependent tropism responses.

### 2.3. Chromosomal Distribution of Cucumber PIN Genes

**Figure 2:**
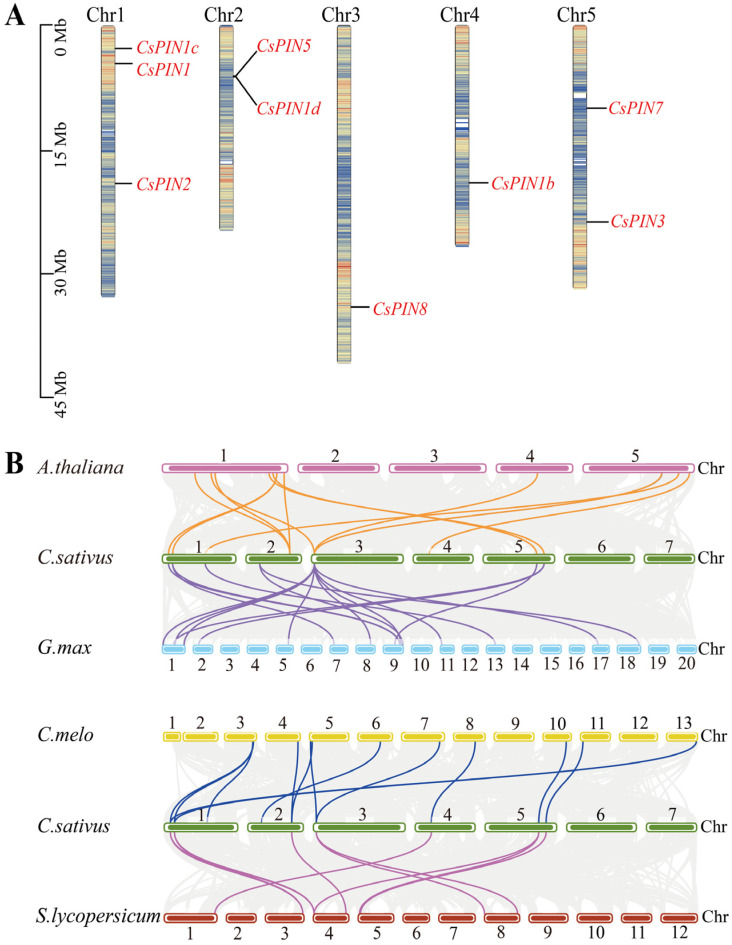
Chromosomal mapping and colinearity analysis of *CsPIN* genes. (**A**) Chromosomal localization. Mb: megabase. The different colors on the chromosome represent the gene density. (**B**) Collinearity analysis. Highlight lines: syntenic *PIN* gene pairs; Gray: collinear blocks.

Chromosome density information was obtained from the genome using the TBtools v 2.119 software, and the positions of the *CsPIN* genes on the chromosomes were located (Figure 2A). The results indicated that nine *CsPIN* genes were unevenly distributed on five chromosomes (Chr1 to Chr5). The chromosome with the highest gene distribution was Chr1 (three genes), followed by Chr2 and Chr5 (two genes each). *CsPIN1d* and *CsPIN5* on Chr2 formed a cluster. Each of Chr3 and Chr4 had one gene.

To further investigate the potential evolutionary relationship of the *CsPIN* genes in cucumber, collinearity analysis was conducted between cucumber and *Arabidopsis*, *G. max*, *Cucumis melo*, and tomato (*Solanum lycopersicum*) (Figure 2B). The results demonstrated that most *CsPIN* genes had at least three pairs of homologous genes in the genomes of other plants. Specifically, *CsPIN1* exhibited the highest number of homologous pairs (seven pairs), followed by *CsPIN1c* (six pairs), *CsPIN1b*, *CsPIN3*, and *CsPIN9* (five pairs each), *CsPIN7* (four pairs), *CsPIN2* and *CsPIN5* (three pairs each), and *CsPIN1d* (one pair). These results indicate that the *PIN* genes in cucumber share a common ancestor with those in other species.

### 2.4. The Conserved Domains and Gene Structure of Cucumber PIN Genes

**Figure 3:**
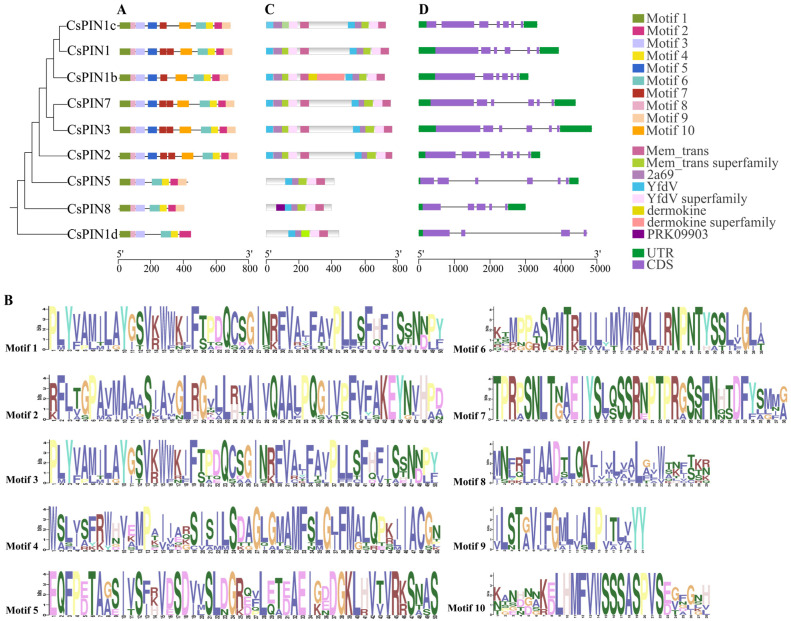
Conserved motifs (**A**,**B**), gene structure (**C**), and exon–intron structure (**D**) of *CsPINs*. Colored boxes of different lengths represent different conserved motifs, and letters in different colors mean the sequences of the conserved motifs.

We further analyzed the conserved domains, gene structure, and exon/intron structure patterns of *CsPINs* (Figure 3). The results revealed that the motif distribution patterns of CsPIN proteins within groups were similar (Figure 3A,B). There are six CsPIN members that contain ten motifs, including CsPIN1, CsPIN1b, CsPIN1c, CsPIN2, CsPIN3, and CsPIN7; CsPIN5 and CsPIN8 contain seven motifs, while CsPIN1d contains six motifs. All nine CsPIN members contain motifs 1, 2, 3, 4, 6, and 8. These results suggest that these motifs are relatively conserved, and some unique motifs may be related to specific functions of the genes.

The Mem_transfamily functional domain was found in the CsPIN family members (Figure 3C). On the other hand, most *CsPIN* genes with similar structures had a similar number of exons/introns but differed in arrangement and length (Figure 3D). For instance, *CsPIN1c* and *CsPIN2* have the largest number of exons, both containing seven exons; *CsPIN1*, *CsPIN1b*, *CsPIN3*, *CsPIN5*, and *CsPIN7* all contain six exons; *CsPIN8* contains five exons; *CsPIN1d* contains the fewest number of exons, with four exons. *CsPIN1d* only contains the upstream non-translated region, excluding the downstream non-translated region. The remaining eight *CsPIN* members all contain 5′ untranslated regions and 3′ untranslated regions. The results indicated that the *CsPIN* genes were relatively conserved during evolution, which ensured the integrity of their gene structure and resulted in minimal functional changes.

### 2.5. Analysis of Cis-Acting Elements Prediction in CsPIN Promoters

**Figure 4:**
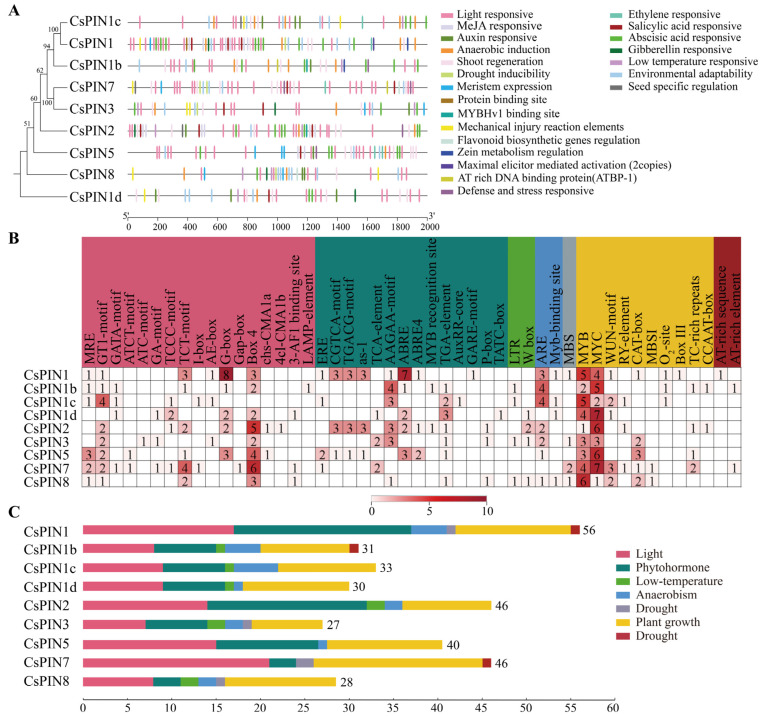
Analysis of cis-acting regulatory elements of the *CsPIN* genes. (**A**) The cis-acting elements distribution in the promoters of *CsPINs*. (**B**,**C**) The names and numbers of cis-acting elements in the promoters of each *CsPIN* gene.

The promoter sequence of the *CsPIN* genes located 2000 bp upstream of coding sequences was analyzed to predict their cis-acting elements (Figure 4A). The results showed that nine *CsPIN* members predicted a total of 48 CAREs, including light response (17), plant hormones (14) (including auxins, gibberellins, salicylic acid, abscisic acid, methyl jasmonate, ethylene), plant growth and development (10), low temperature (2), high temperature (2), drought (1), and anaerobic conditions (1). Among them, cis-acting elements such as light (G-box, GT1 motif, and TCT motif), plant hormones (ABRE, AAGAA motif, and TATC box), and plant growth and development (MYB and MYC) account for the largest proportion in *CsPINs*. *CsPIN7* contains the most light-responsive elements (17) and plant growth and development-related elements (19). *CsPIN1* contained the largest number of elements, with the largest number of plant hormone-related elements (Figure 4B,C). These results indicate that the *CsPIN* gene family is mainly regulated by light and plant hormones.

### 2.6. Interaction Network of CsPIN Proteins

**Figure 5:**
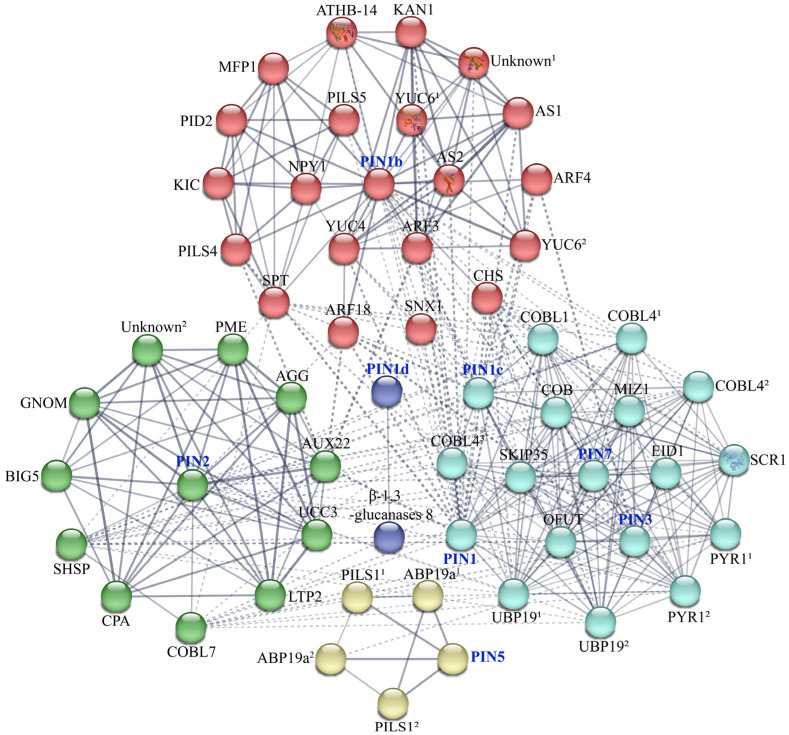
Protein–protein interaction analysis of CsPINs proteins. Abbreviations: ABP19a: auxin-binding protein ABP19a; AGG: agglutinin domain-containing protein; ARF: auxin response factor; AS1: transcription factor AS1; AS2: protein ASYMMETRIC LEAVES 2; ATHB-14: homeobox-leucine zipper protein ATHB-14; AUX22: auxin-induced protein AUX22-like; BIG5: brefeldin A-inhibited guanine nucleotide-exchange protein 5 isoform X1; β-1,3-glucanases 8: glucan endo-1,3-beta-glucosidase 8; CHS: chalcone synthase; COB: protein COBRA; COBL: COBRA-like protein; CPA: F-actin-capping protein subunit alpha; EID1: phytochrome A-associated F-box protein; GNOM: ARF guanine-nucleotide exchange factor GNOM; KAN1: transcription repressor KAN1 isoform X1; KIC: calcium-binding protein KIC; LTP2: non-specific lipid-transfer protein 2; MFP1: MFP1 attachment factor 1; MIZ1: protein MIZU-KUSSEI 1; NPY1: BTB/POZ domain-containing protein NPY1; OFUT: O-fucosyltransferase family protein; PILS: auxin transporter-like protein; PID2: protein kinase PINOID 2; PME: pectinesterase; PYR1: abscisic acid receptor PYR1; SCR1: protein SCARECROW 1; SHSP: SHSP domain-containing protein; SKIP35: ankyrin repeat protein SKIP35; SNX1: sorting nexin 1; SPT: sugar phosphate transporter domain-containing protein; UBP19: ubiquitin-specific protease family C19-related protein; UCC3: uclacyanin-3; Unknown: uncharacterized protein; YUC: indole-3-pyruvate monooxygenase YUCCA. Protein names marked with numbers were assigned based on BLAST alignments (NCBI) to identify entries with identical names but distinct gene IDs.

To predict the interaction between PIN and other proteins in cucumber, we constructed an interaction network using the STRING database (Figure 5). According to the prediction results, we identified eight CsPINs that interact with 34 distinct cucumber proteins. CsPIN may interact with multiple auxin-related proteins, including auxin response factor (ARF), auxin-binding protein (ABP), auxin-induced protein (AUX), serine/threonine protein kinase PINOID (PID), indole-3-pyruvate monooxygenase (YUC), etc. These transcription factors, growth hormone receptors, and auxin transporter proteins work together to participate in the expression of auxin genes, auxin biosynthesis, and transport, and are essential for the formation of tissues and organs such as flowers, stems, and roots.

CsPIN1 and CsPIN1b may interact with transcription factor AS1 (AS1), protein ASYMMETRIC LEAVES 2 (AS2), and transcription repressor KAN1 (KAN1). AS1, a MYB-type transcriptional repressor, regulates leaf development by modulating KNOX gene expression [45]. AS2 is a negative regulator of cell proliferation in the adaxial side of leaves, regulating the formation of the symmetrical lamina and the establishment of venation patterns [46]. It has been reported that AS2 can directly interact with AS1, synergistic with RH10 or RID2 to inhibit the expression of abaxial genes such as ARF3, ARF4, KAN1, and KAN2, and promote adaxial development in leaf primordia at shoot apical meristem under high temperatures, thereby participating in the establishment of leaf polarity [47,48]. Therefore, PIN proteins in cucumber may also regulate the development of narrow leaves by interacting with AS1, AS2, or KAN1.

### 2.7. Expression Profiles of CsPIN Genes in Different Tissues and Organs

**Figure 6:**
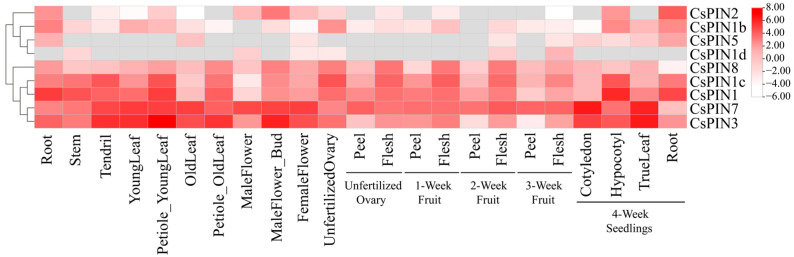
The expression profiles of *CsPIN* genes. The public transcriptome data of 23 cucumber tissues were downloaded, and the *CsPIN* genes heat map were drawn based on the FPKM values of log2.

To elucidate the role of *CsPIN* genes in cucumber growth and development, we obtained RNA sequencing data for *CsPIN* gene expression profiles across 23 distinct tissues from the CuGenDB database v2.0 (biological project PRJNA312872) available at the website (http://cucurbitgenomics.org). As shown in Figure 6, except for *CsPIN1d*, the other eight *CsPIN* genes were all up-regulated in roots, among which *CsPIN1* had the highest expression level in roots. Except for *CsPIN1b*, *CsPIN1d*, *CsPIN2,* and *CsPIN5*, the other five *CsPIN* genes were expressed in all 23 different tissues. *CsPIN1b* was not expressed in the male flowers and pericarp of 2-week-old fruits. *CsPIN1d* was specifically expressed in the stem, male flowers, female flowers, unpollinated ovaries, 2-week-old fruit flesh, and 3-week-old fruit flesh. *CsPIN2* was specifically expressed in the roots, male flowers, male flower buds, female flowers, hypocotyls, and roots of 4-week-old seedlings. *CsPIN5* was specifically expressed in the roots, old leaves, and roots of 4-week-old seedlings. *CsPIN7* was highly expressed in all 23 tissues. Except for *CsPIN1d* and *CsPIN5*, the expression levels of all *CsPIN* genes in young leaves were higher than those in old leaves, indicating that *CsPIN* plays an important role in the early growth and development of leaves. These results indicate that the expression of *CsPIN* genes shows obvious tissue specificity.

### 2.8. Expression Analysis of CsPIN Genes Under Different Stress Conditions

**Figure 7:**
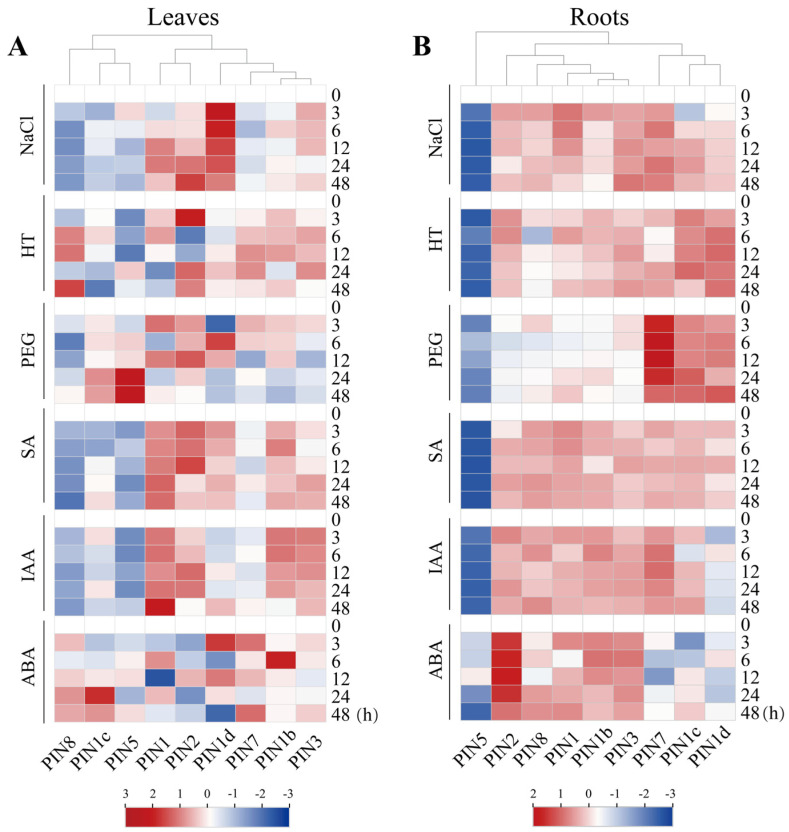
Heatmap of qPCR-based expression patterns of CsPIN genes in cucumber leaves and roots under abiotic stress and hormone treatment. The relative expression level was calculated by 2^−ΔΔCT^ method compared with EF1 alpha (CsaV3_2G011610) as a reference gene.

To explore the expression patterns of *CsPIN* genes under abiotic stresses (NaCl, HT, and PEG) and hormone stresses (SA, IAA, and ABA), we conducted qRT-PCR analysis on cucumber leaves and roots. The results indicated that the expression patterns of *CsPIN* gene family members under different stress conditions were significantly different (Figure 7). Under NaCl treatment, *CsPIN1d* had the highest expression level in leaves, while *CsPIN1b*, *CsPIN1c*, *CsPIN5*, *CsPIN7,* and *CsPIN8* had relatively low expression levels in leaves. Under HT treatment, the expression levels of *CsPIN1*, *CsPIN2*, *CsPIN1b*, *CsPIN3,* and *CsPIN7* were increased in leaves. The expression of *CsPIN5* was suppressed in the roots under NaCl and HT. Under PEG stress treatment, the expression levels of *CsPIN1c*, *CsPIN2*, and *CsPIN5* in leaves were increased; *CsPIN1c*, *CsPIN1d*, and *CsPIN7* were significantly induced in the roots; whereas the the expression levels of *CsPIN1*, *CsPIN1b*, *CsPIN2*, *CsPIN3*, and *CsPIN8* in roots were not significant, with similar patterns of change.

Under SA and IAA hormone stress, the expression patterns of most *CsPIN* genes were similar. In leaves, *CsPIN1* was significantly induced, while *CsPIN1c*, *CsPIN5*, *CsPIN7*, and *CsPIN8* were inhibited; *CsPIN5* was inhibited in roots. After ABA treatment, the expression pattern of the *CsPIN2* gene in leaves and roots showed opposite trends; the expression levels of *CsPIN1b*, *CsPIN1c*, *CsPIN1d*, *CsPIN3*, and *CsPIN8* in leaves increased; *CsPIN1*, *CsPIN1b*, *CsPIN3*, *CsPIN2*, *CsPIN5*, and *CsPIN8* in roots were up-regulated. Under the three hormone treatments, the relative expression levels of most *CsPIN* genes in roots increased. *CsPIN2*, *CsPIN5*, and *CsPIN8* were extremely sensitive to SA, IAA, and ABA. These results indicate that members of the *CsPIN* gene family have different adaptive responses under different abiotic stresses and hormone treatments.

## 3. Discussion

### 3.1. Identification and Evolution of CsPIN Gene Family in Cucumber

Currently, *PIN* gene family members have been identified in multiple species using whole-genome approaches, and the number of the PIN family members varies among different plants. The *PIN* gene family members are quite diverse, with the fewest being four in the with the least number of four members in *Marchantia polymorpha* [49], and relatively large numbers in soybean and wheat [20,50]. In this study, we identified nine *CsPINs* in the cucumber genome (Table 1); this number of *PIN* genes is similar to that in *Arabidopsis* and tomato, suggesting that some *PINs* may have originated from one or more common genes. The expansion of the *PIN* gene family in different species might be attributed to genomic duplication events [16]. Phylogenetic analysis of PIN proteins from five plants classified these proteins into five sub-families (Figure 1), and *CsPINs* have the closest evolutionary relationship with the genes of the dicotyledonous plants soybean and *Arabidopsis*. Multiple gene pairs were also identified with dicotyledonous plants in the collinearity analysis. These findings indicate that these genes likely descended from a common ancestor, and the specific differences of the genes in different species could be attributed to the evolutionary process of plants.

Chromosome mapping analysis revealed that the *CsPIN* genes were significantly unevenly distributed on five chromosomes of cucumber (Figure 2A). Chr3 and Chr4 each had one gene, Chr2 and Chr5 each had two genes, and Chr1 had three genes. Chr2 contained a pair of tandemly duplicated genes, *CsPIN5* and *CsPIN1d*. In addition, *CsPIN1* and *CsPIN1c* were highly similar in gene structure and motifs, indicating that they may have similar functions (Figure 3A). In rice, *OsPIN1a-1d*, *OsPIN3a-3b*, and *OsPIN5a-5c* sequences were similar, suggesting that the *PIN* gene family was generated through chromosomal segment duplication [51]. Maize and wheat also have three and fifteen pairs of repeated genes, respectively [12,50]. The replication and specific amplification of gene fragments throughout the genome have played a significant role in the process of evolution.

To compare the structures of CsPIN proteins, we identified the conserved motifs and domains of CsPIN (Figure 3B,C). We found that all CsPIN proteins contain six conserved motifs (Motif 1-4, Motif 6, Motif 8) and a conserved domain Men_Trans (PF03547). PIN proteins are connected by a heterogeneous central hydrophilic loop between the two highly conserved hydrophobic fragments at the N-terminal and C-terminal [52]. This hydrophilic loop contains four highly conserved sequences (HC1-HC4), among which the long central hydrophilic loop is approximately 300 amino acids, while the short one is about 50–100 amino acids (Figure S1). According to the length of the central hydrophilic loop, PIN proteins can be classified into long or typical PINs, short or atypical PINs, and semi-typical PINs [24,26,53]. In this study, the long and typical members of the cucumber *PIN* gene family include *CsPIN1*, *CsPIN1b*, *CsPIN1c*, *CsPIN2*, *CsPIN3*, and *CsPIN7*; short or atypical PINs are *CsPIN1d*, *CsPIN5*, and *CsPIN8*. Similar to the reported PIN family members in rice, maize, and wheat [12,50,51], there is no homologous gene to *AtPIN6* in cucumber. This result indicates that short or atypical *PINs* evolved independently of long or typical *PINs*. This difference is directly related to the presence or absence of certain motifs in the protein. In *Arabidopsis*, antagonistic interactions between the PID kinase and protein phosphatase 2A (PP2A) dynamically regulate the apical–basal polarity of PIN proteins by modulating their phosphorylation status [54]. PID-mediated phosphorylation of conserved residues within the central hydrophilic loop of PIN proteins (e.g., Ser337/Thr340 in PIN1) promotes their apical membrane localization, whereas PP2A-catalyzed dephosphorylation redirects PINs to the basal membrane [54]. Notably, phylogenetic analysis reveals that all long hydrophilic loop-containing PIN homologs in cucumber retain the conserved Ser337 residue. Furthermore, both CsPIN1 and CsPIN1c exhibit conservation at the Thr340 position (Figure S1), suggesting their potential functional conservation with AtPIN1 in phosphorylation-dependent polar targeting mechanisms.

The diversification of exon–intron structure is believed to have played a significant role in the evolution of certain gene families. The presence of introns promotes exon shuffling, driving gene evolution, allowing the production of multiple proteins from a single gene through alternative splicing, and playing a key role in gene regulation. Through the analysis of the exon–intron structure of nine *CsPIN* gene members, we found that seven of them contain more than six exons (Figure 3D). Homologous genes in the same phylogenetic branch, such as *CsPIN1*/*CsPIN1b* and *CsPIN3*/*CsPIN7*, have similar exon–intron structures. Except for *CsPIN1d*, which shows a significantly different exon–intron structure, the gene structures of other members are relatively conserved. Therefore, we hypothesize that throughout the evolutionary history of cucumbers, *PIN* genes have experienced a series of intron deletions, insertions, and gene duplication events, which may have triggered changes in gene expression patterns and protein functions.

Cis-acting regulatory elements refer to non-coding DNA sequences located in the promoter region of genes. The distribution patterns of different types of cis-acting regulatory elements in the promoter region may reveal differences in gene regulatory mechanisms and functions [55]. In this study, we identified a total of 48 elements, which are mainly involved in light response, hormone response, abiotic stress response, as well as growth and development regulation (Figure 4). Among the nine *CsPIN* genes, there are a total of 60 cis-acting elements related to light response, including MRE, GT1 motif, GATA motif, ATCT motif, ATC motif, GA motif, TCCC motif, TCT motif, etc. At the same time, the *PIN* family also has hormone-induced elements such as auxin, salicylic acid, jasmonic acid, abscisic acid, ethylene, and gibberellin, suggesting that the members of the *PIN* gene family may not only have the biological activity of transporting auxin but also may be involved in the synergistic or antagonistic pathways of different plant hormones and auxin [22]. Similar to the cis-acting elements identified in the promoter region of the coffee PIN gene [56], the promoter regions of *CsPINs* also contain multiple abiotic stress elements, such as LTR and W-box (cold response), myb binding sites (hypoxia induction), MBS (drought induction) elements, etc. These findings suggest that the cucumber *PIN* gene family may play extensive roles in plant responses to abiotic stress; however, further experimental validation is required to confirm their precise regulatory mechanisms.

### 3.2. Protein–Protein Interaction Network of CsPIN

Numerous studies have found that protein–protein interactions can accurately predict the cellular functions of uncharacterized proteins [57]. In this study, eight CsPIN proteins were predicted to interact with 34 kinds of proteins (Figure 5). We predicted that CsPIN2 in cucumber might interact with GNOM and BIG5 (BEN1). We also found that five CsPIN protein members (PIN1, PIN1b, PIN1c, PIN2, and PIN3) in cucumber may interact with ARF and USP19 (Figure 5). In *Arabidopsis*, GNOM, as a membrane-associated guanine nucleotide exchange factor on ADP-ribosylation factor G protein (ARF GEF), regulates the vesicle transport required for the polar localization of auxin efflux carriers, thereby determining the direction of auxin flow [58]. It has been reported that GNOM mediates the sorting of PIN1 from endosomal compartments to the basal PM and the polarization of PIN3 to the bottom side of hypocotyl endodermal cells in the hypocotyl [59,60]. BEN1 and BEN2 play essential roles in the polar localization of PIN, dynamic repolarization events, and the establishment of auxin activity gradients [61]. These processes are vital for various developmental mechanisms, such as embryonic pattern formation, organogenesis, and vascular venation pattern formation [61]. It has been reported that four VvPIN protein members in grape may interact with ARF to regulate plant growth and development processes by controlling auxin response genes [15]. It has been reported that auxin can regulate the transcription of multiple AtPIN proteins in a tissue-specific manner through the TIR1-Aux/IAA-ARF pathway [62]; at the same time, auxin may also regulate the protein stability of AtPIN2 through the mechanisms of ubiquitination and proteasome activity [63]. In this study, CsPIN1b may interact with CsPID2, and at the same time, CsPIN1 and CsPIN1b may interact with multiple proteins involved in leaf morphology (transcription factor AS1, ASYMMETRIC LEAVES 2, and transcription repressor KAN1) (Figure 5). PID has been reported to be capable of catalyzing the phosphorylation of PIN and plays a key role in regulating the apical–basal PIN polarity [64]. In wheat, TaPIN8 and TaPIN9 may interact with TaPID under drought and heat stress conditions, influencing the localization and polar auxin transport of TaPIN by regulating the phosphorylation state [50]. Auxin is also crucial for regulating leaf development. Under warm conditions, the photoreceptor phytochrome-interacting factor 4 (PIF4) directly activates the protein kinase PID, promoting auxin production in leaves and leading to auxin accumulation in petioles [65]. At the same time, PID polarizes the auxin transporter PIN3 to the outer membrane of petiole cells through phosphorylation. Moreover, AS1 mainly regulates the induced expression of PID on the back of the petiole, indicating that the polar transport of auxin is a key biochemical event in the process of leaf temperature regulation [65]. The triple mutants of ospin1c ospin1d ospid have also been reported to have serious defects in leaf morphogenesis in rice [66]. While these findings advance our understanding of auxin-mediated co-regulation of PIN polar transport in cucumber, future studies should employ co-immunoprecipitation (Co-IP) and yeast two-hybrid (Y2H) assays to experimentally map the interaction networks among these signaling components.

### 3.3. Role of the CsPIN Genes in Plant Growth and Development

The gene functions of the *PIN* gene family members exhibit tissue specificity, and this differentiated expression pattern plays a significant role in the growth and development of cucumbers. In this study, we obtained RNA-Seq data for 23 different cucumber tissue expression profiles from the CuGenDB database and examined the transcriptional levels of nine *CsPIN* genes. The results showed that *CsPIN* genes from different phylogenetic branches exhibited multiple expression patterns in different organs (Figure 6). In Group I, *CsPIN2*, *CsPIN1b*, and *CsPIN5* had similar expression patterns, mainly expressed in roots. *CsPIN1d* was expressed in stems but not in other organs. The five genes (*CsPIN1*, *CsPIN1c*, *CsPIN3*, *CsPIN7*, and *CsPIN8*) in Group II were highly expressed in roots, stems, tendrils, and petioles of new leaves. Among them, *CsPIN1* had the highest expression levels in mature roots and four-week-old hypocotyls, and weak expression in male flowers and old leaves, while *CsPIN3* and *CsPIN7* were highly expressed in male flowers, four-week-old cotyledons, and true leaves. Similar expression patterns have also been found in other species. In *Arabidopsis*, *AtPIN1* and *AtPIN3* display substantial expression levels in root tissues, influencing the dimensions of the primary root meristem and the growth rate of the primary root [67]. *GbPIN1*, *GbPIN2*, and *GbPIN3* exhibit substantial expression levels in the roots and stems of cotton but are nearly absent in leaves [68]. *PbPIN3-1*, *PbPIN3-2*, *PbPIN3-3*, and *PbPIN4* display similar expression patterns across various organs in both dwarfing (QN101) and vigorous (OHF51) pear rootstocks, suggesting functional redundancy among these genes [16]. In rice, *OsPIN5a* and *OsPIN5c* show high expression in leaves, shoot tips, and panicles, whereas *OsPIN5b* is predominantly expressed in young panicles [38]. *TaPIN5*, *9*, *13*, *21*, and *28* are highly expressed in wheat stems, while *TaPIN31*, *32*, *35*, *40*, and *44* exhibit elevated expression levels in grains [50]. In maize, most *PIN* gene members are highly expressed in embryos, roots, and stems, with *ZmPIN1b* showing significant expression during female inflorescence development [41]. In tobacco, multiple *NtPIN* genes are highly expressed in stems, and *NtPIN5a* and *NtPIN5b* have higher expression levels in flowers [19]. The above research results indicate that roots, axillary buds, and young stems are the main regulatory targets of PIN proteins [69]. Members of the *PIN* gene family regulate the distribution of auxin through a coordinated and redundant mechanism, playing a crucial role in the normal growth and development of plants [14,18].

The *PIN* gene facilitates plant adaptation to adverse environmental conditions through the regulation of auxin distribution. In cucumber leaves, the transcription levels of *CsPIN* genes under abiotic stress and hormone treatments were significantly different (Figure 7). Among them, *CsPIN1*, *CsPIN1d*, *CsPIN2*, and *CsPIN3* were all induced under NaCl treatment. The expression levels of *CsPIN1c* and *CsPIN5* under PEG treatment were significantly higher than those of other *CsPIN* genes. Four genes (*CsPIN1*, *CsPIN1b*, *CsPIN2*, and *CsPIN3*) were significantly induced under SA and IAA treatments. Meanwhile, the above- and below-ground parts had opposite expression patterns. Except for *CsPIN5*, most *CsPIN* genes in roots were induced under both abiotic and hormone stresses. *CsPIN2* and *CsPIN7* were sensitive to PEG and ABA treatments, respectively. Several *PIN* genes in grapes, soybeans, and maize respond to different abiotic or hormone stresses [15,20,41]. *VvPIN7* and *VvPIN9* are sensitive to PEG treatment [15]; *GmPIN* genes can be induced by various abiotic stresses and plant hormones, among which the expression level of *GmPIN5a* is inhibited after IAA treatment [20]; the expression levels of *ZmPIN5c*, *ZmPIN15*, and *ZmPIN10b* are all up-regulated under NaCl treatment [41]. *PIN* genes are highly expressed in cotton plants under drought, salt, and dehydration treatments [18]. These findings suggest that by modulating the expression levels of *PIN* genes, the dynamic equilibrium of auxin in various tissues and organs is maintained, thereby contributing to tissue and organ formation and differentiation. Future studies employing CRISPR/Cas9 knockout technology will be essential to elucidate the precise spatiotemporal roles of *CsPIN* genes in developmental processes such as phyllotaxis and root gravitropism.

## 4. Materials and Methods

### 4.1. Identification and Physicochemical Properties of PIN Genes in Cucumber

To identify the *PIN* genes in cucumber (Chinese Long), the whole genome sequences were retrieved from the Cucurbit Genomics Database (CuGenDBv2) website (http://cucurbitgenomics.org/v2/, accessed on 8 October 2024). *Arabidopsis* PIN protein sequences were obtained from TAIR 10 (https://www.arabidopsis.org/, accessed on 8 October 2024). Next, the Hidden Markov Model (HMM) of the PIN domain (PF03547) was obtained from the Pfam database (http://pfam.xfam.org/, accessed on 8 October 2024), and the HMMER 3.0 software was used to search for candidate *PIN* genes. Then, redundant sequences were removed to ensure only unique candidate *CsPIN* genes were retained. Using ExPASy online (https://web.expasy.org/protparam/, accessed on 19 October 2024) program to analyze the physicochemical properties of cucumber PIN protein. Subcellular locations of CsPIN proteins were predicted using the LocTree3 server (https://rostlab.org/services/loctree3/, accessed on 24 October 2024).

### 4.2. Phylogenetic Analysis of Cucumber PIN Gene Family

MEGA6 software was used to perform multiple sequence alignment of PIN protein sequences in cucumber, *Arabidopsis*, wheat, soybean, and rice species. And the multi-sequence alignment result file was converted into meg format for PIN protein phylogenetic analysis.

### 4.3. Chromosome Localization and Gene Duplication Analysis

TBtools v 2.119 software was used to obtain chromosome density information from genome annotation, and the chromosomal distribution, length, and the start and end positions of *CsPIN* genes were screened for and located as *CsPINs* according to their distribution on chromosomes. Then the visualization analysis was performed using the Gene Location Visualize from the GTF/ TFF function. Gene annotation files and genome files for *Arabidopsis*, *G. max*, *C. melo*, and *S. lycopersicum* were downloaded using the Ensembl Plants database (https://plants.ensembl.org/index.html, accessed on 22 November 2024).

Used one-step McScanx-super-fast to map chromosome location. The results of the blast were simplified by using the Text merge for MCScanX function, and TBtools Multiple Synteny Plot function was used to highlight the identified PIN collinear pairs and their collinear pairs with the other four species.

### 4.4. The Conserved Motifs, Gene Structure, Function Domain, Putative Cis-Acting Elements Analysis

The gff3 annotation file (ChineseLong_v3.gff3.gz), the genome file (ChineseLong_genome_v3.fa.gz), the gene CDS sequence file (Chinese Long_CDS_v3.fa.gz), and the protein sequence file (ChineseLong_pep_v3.fa.gz) were downloaded from the cucumber database. The MEME website (https://meme-suite.org/meme/, accessed on 21 November 2024) was used on the cucumber PIN family to predict the conservative base sequence of the protein sequence; the largest number of motifs was set to 10, and the rest of the parameter was set to the default value. Through a CD search on the NCBI website (https://www.ncbi.nlm.nih.gov/cdd, accessed on 26 November 2024), conservative domain analysis of the cucumber PIN protein sequence was performed. Exon–intron structure prediction and gene structure analysis of *CsPIN* genes were performed at the GSDS online website (https://gsds.gao-lab.org/, accessed on 25 November 2024). Ultimately, they were further visualized through the Gene Structure View function in TBtools.

The promoter sequences (2000 bp before the start codon of a gene) were extracted from the cucumber genome database. These sequences were subsequently submitted to PlantCARE (http://bioinformatics.psb.ugent.be/webtools/plantcare/html/, accessed on 7 November 2024). The cis-acting elements were analyzed, and the predicted results were submitted to the TB tool software for visual analysis.

### 4.5. Protein–Protein Interaction Network

The functional interaction network model of CsPIN proteins was established by using the STRING database (https://cn.string-db.org/, accessed on 10 November 2024) to predict the relationship between CsPIN proteins and other related proteins. The species was designated as Cucumis sativus, with confidence parameters set to a threshold of 0.40 and disconnected nodes hidden in the network.

### 4.6. Analysis of Expression Profiles of CsPINs Genes in Different Tissues

To examine the tissue-specific expression patterns of *CsPIN* genes, we retrieved expression data with accession number PRJNA312872 from the Cucurbit Genomics Database (http://cucurbitgenomics.org/v2/download, accessed on 11 November 2024). A total of 23 different tissues and organs of cucumber were downloaded with FPKM transcriptome data, including roots, stems, tendrils, young leaves, young leaf petioles, old leaves, old leaf petioles, male flowers, male flower buds, female flowers, unfertilized ovary, unfertilized ovary peels, unfertilized ovary fleshes, one week fruit peels, one week fruit flesh, two week fruit peels, two week fruit flesh, three week fruit peels, three week fruit flesh, four week old cotyledon, four week old hypocotyls, four week old true leaves, and four week old roots. Meanwhile, the FPKM values were converted using the log2 method, and the *CsPIN* gene expression heatmap was drawn using TBtools software.

### 4.7. Plant Materials and Treatment

The cucumber cultivar “Chunqiu Wang No. 3” was used as an experimental material and planted in the Chongming Base of National Engineering Research Center for Facility Agriculture, Shanghai Academy of Agricultural Sciences (Shanghai, China). Cucumber seedlings were grown in a plant growth chamber (26 °C/18 °C day/night condition, 14/10 h (light/darkness) photoperiod, 75% relative humidity). When the seedlings reached a true leaf stage, the root of plantlets was soaked in a hydroponic nutrient solution, and an oxygen pump was added to the basin containing the nutrient solution to ensure an adequate oxygen supply. Uniformly grown three-week-old cucumber seedlings were selected and treated according to previously published concentrations for plant hormones and abiotic stresses [11,15,50,70,71], including 1 mmol/L IAA, 5 mmol/L SA, 100 μmol/L ABA, 150 mmol/L NaCl, 5% PEG 6000, and high temperature (35 °C). Leaf and root samples were collected at 0, 3, 6, 12, 24, and 48 h after treatment. All samples were immediately immersed in liquid nitrogen and stored at −80 °C. Three biological replicates were set for each treatment.

### 4.8. RNA Extraction and Quantitative qRT-PCR Analysis

Total RNA was extracted from the treated and control samples in the leaves and roots of cucumber using a high-purity total RNA rapid extraction kit (Takara Biomedical Technology Co., Beijing, China). The quality and concentration of different RNA samples were quantified using a NanoDrop (ND-1000, NanoDrop Technologies, Wilmington, DE, USA), followed by reverse transcription using Prime Script RT reagent kit (Takara, Beijing, China) according to the manufacturer’s instructions. The qRT-PCR reactions were performed using the QuantStudio 6 Flex Real-Time PCR System (Thermo Fisher Scientific, Waltham, MA, USA). Gene-specific primer pairs for all *CsPIN* genes were designed using NCBI Primer-BLAST with default parameters (Table S1). The amplification was carried out as follows: 95 °C for 5 min, 45 cycles at 95 °C for 10 s, 55 °C for 20 s, and 72 °C for 20 s, followed by a dissociation stage. The relative expression levels of CsPINs were calculated using the 2^−ΔΔCT^ method. All the expression analyses included three biological replicates and three technical replicates.

## 5. Conclusions

In general, this study identified nine *CsPIN* family members and systematically analyzed their genomic locations, phylogenetic relationships, conserved domains, gene structures, protein interactions, and gene expression levels. Meanwhile, *CsPIN* genes were significantly differentially expressed during the development of various cucumber tissues, with specific members playing leading roles in regulating root polarity development and leaf morphogenesis. In response to abiotic stresses such as NaCl, HT, and PEG, as well as various hormone stimuli, different *CsPIN* genes exhibited diverse response patterns, coordinating the dynamic balance of auxin to adapt to environmental changes. However, the details of functional redundancy among cucumber *PIN* genes and their complex interaction networks require further elucidation. In the future, we plan to utilize single-cell sequencing and gene editing technologies to further explore the functions of *CsPIN* genes, providing key targets for molecular breeding and targeted genetic improvement of cucumber growth and development.

## Figures and Tables

**Table 1:** Characteristics of the putative PIN-FORMED (PIN) proteins in cucumber.

Gene Name	Gene Locus	Chromosome Location	ORF Length (bp)	No. of AA	MW (kDa)	pI	Aliphatic Index	GRAVY	TMHs	Subcellular Localization ^#^
CsPIN1	CsaV3_1G007160.1	chr1:4542181–4546111(−)	1857	618	67.44	9.13	89.61	0.077	9	PM
CsPIN1b	CsaV3_4G029470.1	chr4:19012575–19015651(+)	1791	596	63.70	8.77	96.71	0.264	9	ER
CsPIN1c	CsaV3_1G004350.1	chr1:2730713–2734038(−)	1827	608	66.63	9.09	90.90	0.098	8	PM
CsPIN1d	CsaV3_2G009700.1	chr2:6188991–6193716(+)	1191	396	43.04	7.57	112.75	0.494	5	ER
CsPIN2	CsaV3_1G032010.1	chr1:19030115–19033521(+)	1938	645	70.83	9.29	85.46	0.017	9	PM
CsPIN3	CsaV3_5G028620.1	chr5:23739663–23744519(−)	1911	636	69.46	7.12	91.42	0.111	9	PM
CsPIN5	CsaV3_2G009610.1	chr2:6112103–6116586(+)	1116	371	40.37	7.04	112.24	0.675	9	ER
CsPIN7	CsaV3_5G013380.1	chr5:9982996–9987398(−)	1890	629	68.48	8.50	92.62	0.137	9	PM
CsPIN8	CsaV3_3G041710.1	chr3:34023847–34026847(+)	1071	356	38.98	9.59	129.61	0.692	8	ER

(+): forward strand; (−): reverse strand; bp: base pair; AA: amino acid; MW: molecular weight, kDa: kilodalton; pI: isoelectric point; GRAVY: grand average of hydropathicity; TMHs: transmembrane helices; #: based on the prediction of the LocTree3 website; PM: plasma membrane; ER: endoplasmic reticulum membrane.

## Data Availability

The original contributions presented in this study are included in the article/Supplementary Material. Further inquiries can be directed to the corresponding author.

## Supplementary Materials

The following supporting information can be downloaded at https://www.mdpi.com/article/10.3390/plants14111566/s1, Figure S1: Multiple sequence alignments of PIN domains. Table S1: Primers for RT-qPCR analysis of *PIN* genes in cucumber.

## Abbreviations

The following abbreviations are used in this manuscript:

ABA	Abscisic acid
HT	High temperature
IAA	Indole-3-acetic acid
NaCl	Sodium chloride
PEG	Polyethylene glycol
SA	Salicylic acid
qRT-PCR	Quantitative real-time polymerase chain reaction
